# Extraction of Energy Characteristics of Blue Whale Vocalizations Based on Empirical Mode Decomposition

**DOI:** 10.3390/s22072737

**Published:** 2022-04-02

**Authors:** Chai-Sheng Wen, Chin-Feng Lin, Shun-Hsyung Chang

**Affiliations:** 1 Department of Electrical Engineering, National Taiwan Ocean University, Keelung 20224, Taiwan; 21053001@mail.ntou.edu.tw (C.-S.W.); lcf1024@mail.ntou.edu.tw (C.-F.L.); 2 Department of Microelectronics Engineering, National Kaohsiung University of Science and Technology, Kaohsuing 81157, Taiwan

**Keywords:** blue whale vocalizations, empirical mode decomposition, energy ratio, energy density intensity

## Abstract

This study extracts the energy characteristic distributions of the intrinsic mode functions (IMFs) and residue functions (RF) for a blue whale sound signal, with empirical mode decomposition (EMD) as the basic theoretical framework. A high-resolution marginal frequency characteristics extraction method, based on EMD with energy density intensity (EDI) parameters for blue B call vocalizations, was proposed. The extraction algorithm included six steps: EMD, energy analysis, marginal frequency (MF) analysis with EDI parameters, feature extraction (FE), classification, and Hilbert spectrum (HS) analysis. The blue whale sound sources were obtained from the website of the Scripps Whale Acoustics Lab of the University of California, San Diego, USA. The source is a type of B call with a time duration of 46.65 s, from which 59 analysis samples with a time duration of 180 ms were taken. The average energy distribution ratios of the IMF1, IMF2, IMF3, IMF4, and RF are 49.06%, 20.58%, 13.51%, 10.94% and 3.84%, respectively. New classification criteria and EDI parameters were proposed to extract the blue whale B call vocalization (BWBCV) characteristics. The analysis results show that the main frequency bands of the signal are distributed at 41–43 Hz in the MF of IMF1 for Class I BWBCV and 11–13 Hz in the MF of IMF2 for Class II BWBCV, respectively.

## 1. Introduction

The blue whale (Balaenoptera musculus) [1] is a marine mammal belonging to the order of the baleen whale. It is not only the largest animal in existence on Earth, but also may be one of the largest animals in the history of the Earth, with a body length of up to 30 m, and a weight of approximately 80 to 150 metric tons. Blue whales have an average normal life expectancy of 80 to 90 years, and forage at diving depths of up to 300 m [2,3]. Their range of activities alters with changes in the seasonal climate food chain and global sea area. Currently known subspecies have their habitats mainly in the North Atlantic Ocean extending into the Greenland Sea, the northeastern Pacific Ocean along the west coast of the United States to Mexico, the northern Indian Ocean near the sea, and located in the southern hemisphere at a higher latitude than the Antarctic blue whale. Pygmy blue whales are most abundant in the waters off Australia and New Zealand. However, in the mass whaling years before the early 20th century, the commercial behavior of whalers made blue whales almost extinct, and they became an endangered species. In the 1960s, the international community’s attention to whale conservation led to the beginning of a ban on whaling, so the global whale population increased and is currently high [4,5].

Blue whales use their special physiological structure to make sounds to communicate their underwater activities between whales. The information exchanged includes identification, foraging, warning, courtship, environmental information transmission, and geomorphological location positioning. Its voice is very loud, has a low frequency, can reach more than 800 km away, and contains strong harmonic signal content. Cummings et al. [6] asserted that the magnitude of the sound of blue whales could reach 155 to 188 dB at the source, with a base frequency of approximately 10 to 40 Hz, and a sound duration of approximately 10 to 30 s. McDonald et al. [7] summarized the types of sound sources in 2001 from A-B-C-D calls based on blue whale sound data observed on the west coast of California, United States of America, and the northeast Pacific. Blue whales have unique calls and significant variability among their populations. Type A calls show a variability of 19 to 23 pulses between 92 and 85 Hz for the prominent overtone. The fast Fourier transform (FFT) length is 0.5 s and the overlap is 87.5% with a Hann window used for the signal analysis. Mellinger et al. [8] studied the sound modalities of blue whales in the North Atlantic in 2003, and found sounds within a sequence composed of two-part phrases repeated every 73 sec, with a phrase exhibiting a constant-frequency tonal of 1–20 Hz and lasting approximately 24 s. Stafford et al. [9] observed the sound patterns of blue whales with the change in regions and seasons. They reported that different vocalization types may represent at least two distinct groups of blue whales in the North Pacific; vocalization A lasted 18.2 s with a frequency of 15.3 Hz, vocalization B swept from 18 to 16.1 Hz for 17.5 s, and the mean time between the A and B vocalizations was 25.6 s. Wiggins et al. [10] observed diel and seasonal calling patterns for blue whales, and found that the night time calling was greater than daytime calling. They produced a spectrogram of blue whale pulsed and tonal down-swept calls at frequencies of 15–35 Hz, which showed a series of harmonically related nearly tonal, linear down-sweeps that started at 52.0 Hz, and ended 10 s later at 47.9 Hz. These studies almost exclusively used FFT to generate the spectrograms. Bouffaut et al. [11] proposed a stochastic matched file to detect the sound of Antarctic blue whales. The stochastic matched file was derived from the matched filter to maximize the signal to noise ratio (SNR) of stochastic signals embedded in colored noise, known as a Z call, in which the pulse is between 15 and 30 Hz, and has an approximate duration of 26.2 s.

A powerful underwater acoustic detection and classification system can obtain the sounds of marine cetaceans in an unsteady and diverse underwater signal environment, understand the changes in cetacean vocality, and study why cetaceans make sounds and what their purposes are. In recent years, the global development of machine learning methods and automation technology has led to improvements in the following areas of research [12,13]: signal data recording and pre-processing [14], extracting signal characteristics [15], signal detection analysis [16], and signal classification dataset systems [17]. Underwater signals obtained through high-sensitivity underwater microphones include environmental, human-made, and marine life sounds. However, many of these sounds have not dealt with cetacean sounds. Therefore, the selection of the algorithm and characteristic parameters that are relevant to the desired signal in the acquired data is of great importance in order to improve the accuracy and integrity of the signal detection and classification. Many methods can be used to extract signal characteristics, such as short time Fourier transform, Wigner Ville distribution functions [18], wavelet transforms (WT), and time–frequency distribution functions (TFD). There are also detailed studies on the family of Cohen time–frequency distribution functions [19], Hilbert Huang transforms (HHT), empirical mode decomposition (EMD) [20], linear prediction coefficients [21], and mel-scale frequency cepstral coefficients [22], etc.

HHTs were proposed by Huang of the Central Research Institute in 1996 [23], which are based on the well-known Hilbert transform (HT), and can be applied to nonstationary and nonlinear signal analysis using the sifting process of EMD. The signal can be disassembled into a multiple time-domain intrinsic mode function (IMF) and a residue function (RF). Thus, the HT can be used to calculate the instantaneous frequency (IF) of each IMF and RF. The HHT algorithm is quite different from the traditional TFD, as the process does not use the kernel function as a basis and unlike the TFD, it is not limited by the length of time of the sampling points per function conversion. Because of this, the HHT does not suffer from a decrease in IF resolution, and it can correct the real-time reaction time and frequency spectrum relationship. In recent years, accurate results obtained from the application of HHT have been reported. Adam et al. [24] introduced the HHT as an effective method for the analysis of marine bio-acoustical signals, to compare the results obtained from three TFDs: the Fourier spectrogram, WT, and HHT. The results show that HHT is a viable alternative to the Fourier and wavelet transforms. Oliver et al. [25,26] then used the HHT theory to propose innovative research methods for the vocalization analysis of cetacean killer whales and sperm whales.

The authors reported that when the SNRs are low or multiple vocalizations are simultaneously emitted in a natural environment, it creates issues in the application of the method. The advantage of EMD properties, in which the successive IMFs represent the original data broken down into frequency components from the highest to lowest frequency, can help HHTs to overcome these challenges. The results confirm that these methods are favorable alternatives for the feature extraction of cetacean vocalizations. Oestreich et al.’s [27,28] visuals show the frequency spectrum of the B call (fundamental frequency) and second through fifth harmonics using the Fourier spectral analysis method. However, they do not reveal the numericalization frequency distribution ranges of the B call (fundamental frequency) and second through fifth harmonics. Therefore, in this study, we continue the sound source characteristic analysis for marine whale species [29] and extend the research of [30] for IMFs and RF energy distribution characteristic analysis technology for blue whale vocalizations. In terms of the innovative contributions, the novelty of this research is in the energy characteristic analysis method proposed to analyze the signal characteristics using the EMD method with the six main steps of the feature extraction algorithm: EMD, energy analysis, MF, feature extraction (FE), classification by EDI, and HS analysis. The proposed method is based on the EMD method applied to the energy characteristics and time–frequency and energy distribution spectrum analysis of blue whale sound signals. It can obtain the signal characteristic information of underwater sound diversity and provide key insights for marine life, underwater acoustics, defense science, and technological research.

The rest of this paper is organized as follows. Section 2 introduces the energy characteristic analysis method, which consists of analyzing the IF of each IMF, energy ratio, frequency feature extraction, the marginal frequency (MF), Hilbert spectrum (HS), and energy density intensity (EDI) parameters. Section 3 presents the blue whale sound signal energy characteristics and spectrum analysis of the experimental results. Section 4 discusses the results and proposes future developments, and Section 5 concludes the paper.

## 2. Energy Characteristic Analysis Method

The IF is an important signal characteristic parameter in the TFD function, and analyzing how it changes over time will be a necessary process in designing the ocean whale and dolphin sound signal acquisition analysis algorithm. This means that the transient frequency of a stationary sine wave signal falling within each sampling time interval is a fixed value, that is, containing only one frequency at a certain time, or frequency changes are limited to a very small interval. However, for the multi-component signals of nonstationary underwater sound in the ocean, the average IF of solving a single value does not conform to the characteristics of the actual signal in a practical physical sense. Therefore, disassembling the signal components improves the resolution of the signal, and analyzing the time, frequency, and energy distribution of each signal component is an important topic in this study.

If these signal parameters are time signals without any restrictions, the calculated IF may not be the correct result for the real signal. Therefore, a variety of modified time–frequency distribution analysis methods can obtain the correct solution for these nonstationary signals. Among them, the HHT time–frequency analysis method can be used for multiple nonstationary signal analysis. Using the sifting process of the EMD method, the original signal can be disassembled into *N* IMFs and one RF; recombination of each IMFs and RF will return the original signal, and this is called the reconstruction process. *Z*(*t*) is the blue whale B call vocalization (BWBCV) sample with a time duration of 180 ms, and is given by:

(1) $$Z\left(t\right)=\overset{N}{\underset{i=1}{\sum }}{\mathrm{IMF}}_{i}\left(t\right)+rf\left(t\right)\;\;$$

IMF_*i*_(*t*) is the *i*th IMF, and *rf*(*t*) is the RF. After the HT, the original signal can be represented by the real and imaginary parts of each IMF as follows:

(2) $$Z_{i}\left(t\right)={\mathrm{IMF}}_{i}\left(t\right)+j\mathrm{HT}\{{\mathrm{IMF}}_{i}\left(t\right)\}=a_{i}\left(t\right)e^{j\theta_{i}\left(t\right)}$$

where a*_i_*(*t*) is the amplitude size. ω*_i_*(*t*) is the angular frequency,

(3) $$\omega_{i}\left(t\right)=\frac{d\theta_{i}\left(t\right)}{dt},\theta_{i}\left(t\right)=arctan\left[\frac{\mathrm{HT}\{{\mathrm{IMF}}_{i}\left(t\right)\}}{{\mathrm{IMF}}_{i}\left(t\right)}\right]\;\;\;$$

Therefore, the IF of the *i*th IMF can be calculated as:

(4) $$\;{\mathrm{IF}}_{i}\left(t\right)=\frac{1}{2\pi }\frac{d\theta_{i}\left(t\right)}{dt}$$

**Figure 1:**
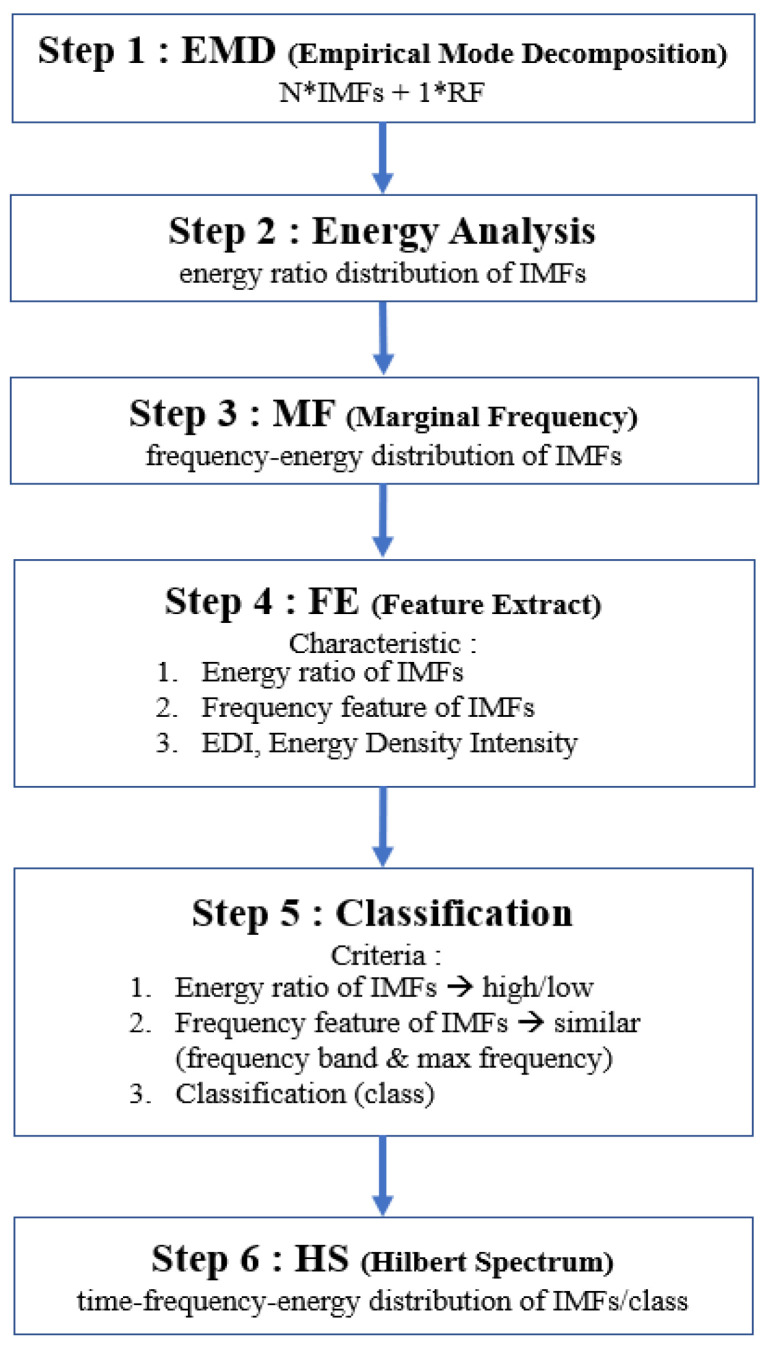
Feature extraction algorithm with six main steps.

In this study, the energy characteristic analysis method is proposed to analyze the signal characteristics using the EMD method. Figure 1 shows the feature extraction algorithm with six main steps: EMD, energy analysis, MF, feature extraction (FE), classification by EDI, and HS analysis.

(1) Empirical Mode Decomposition (EMD)

In underwater acoustic signal processing technology, signal data recording and pre-processing are usually carried out by a passive acoustic monitoring system (PAM). Underwater vocalizations of marine life (e.g., click, whistle, and bust pulse) are extracted from the audio signal by signal feature extraction. The total length of time of the original vocalizations and the sampling frequency of the vocalization signal can be obtained. Using the EMD method in the time–frequency analysis, each sampling time interval signal is disassembled into *N* IMFs and one RF.

**Figure 2:**
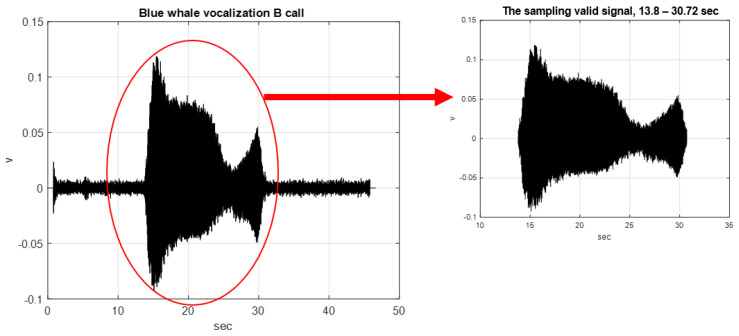
Blue whale B call vocalization with noise (the time length is 46.65 s, sampling frequency is 7350 Hz), and sampling valid signal (the time length is 16.92 s).

This study analyzes the blue whale sound source provided by the website of the Scripps Whale Acoustics Lab of the University of California, San Diego, USA [31]. The sound source type was a B call vocalization with noise, and duration of 46.65 s. A time length of 16.92 is available for valid signal analysis, and the sampling frequency of the B call vocalization was 7350 Hz, as shown in Figure 2.

**Figure 3:**
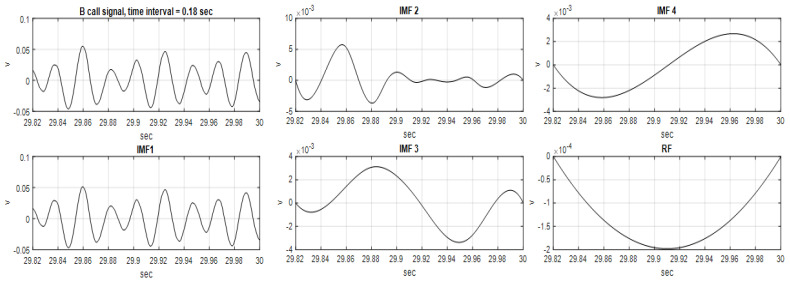
Four IMFs and one RF of one of the 59 BWBCV analysis samples, with a time interval of 29.82 to 30.00 s.

A total of 94 BWBCV analysis samples with a time duration of 180 ms were extracted from the blue whale B call vocalization. Among the 94 sampling signals, after EMD disassembly signals, 6 sampling signals were obtained for six IMFs and one RF, 17 sampling signals for five IMFs and one RF, and 59 sampling signals for four IMFs and one RF were obtained; 12 sampling signals for three IMFs and one RF were also obtained. Selecting the same number of IMF as the signal classification characteristic analysis is the first important criterion for this algorithm process; therefore, 59 sampling signals with four IMFs and one RF with nearer signal characteristics are used as the main signal samples in the paper. Figure 3 illustrates four IMFs and one RF of one sample with a time interval of 29.82 to 30.00 s.

(2) Energy Analysis

After the reconstruction process using the EMD, the original BWBCV signal can be reconstructed from the sum of all IMFs and RF, and each IMF can be regarded as a separate signal function. Therefore, the total energy is defined as the sum of the energies of all IMFs and the RF:

(5) $$E_{total}=\overset{N}{\underset{i=1}{\sum }}{\mathrm{IMF}}_{i}^{2}\left(t\right)+rf^{2}\left(t\right)$$

The energy ratio of the *i*th IMF is:

(6) $$ENGIMF_{i}=\frac{{\mathrm{IMF}}_{i}^{2}}{E_{total}}\times 100\%$$

The energy ratio of the RF is:

(7) $$ENGrf_{i}=\frac{rf_{i}^{2}}{E_{total}}\times 100\%$$

As stated above, the energy ratio is the first very important feature parameter of this analysis method, from which the energy intensity change of the signal in the time and frequency domains can be estimated. The next step performs feature extraction. As shown in Table 1, the average energy ratios of the IMF 1/2/3/4/RF were 49.06%, 20.58%, 13.51%, 10.94%, and 3.84%, respectively, in the 59 samples.

(3) Marginal Frequency (MF)

Following the above steps, all IMFs are available for the HT operation, and the angular frequency of the signal has been obtained. After the angular frequency is calculated, the IF function *IFi*(*t*) of this IMF can be obtained. The MF of the *ith* IMF (average frequency–energy distribution) is defined as follows with a sampling frequency range of *m*–*n* Hz:

(8) $$MFi=\frac{{\mathrm{IMF}}_{imn}^{2}\left(f\right)}{E_{total}}\times 100\%$$

the MF of the RF (average frequency–energy distribution) is:

(9) $$MF_{rf}=\frac{rf_{mn}^{2}\left(f\right)}{E_{total}}\times 100$$

(4) Feature Extraction (FE)

We define the energy concentration frequency band correlation of the IMF, as R[x], $R_{i}\left[x\right]=MFi-\Delta$, where *x* is the frequency between the sampling resolution m and n Hz, Δ is a threshold parameter of the average energy ratio distribution of the IMF, and MFi is the energy marginal spectrum of the m-*n* Hz frequency band for the *i*th IMF as above. Setting the threshold of the average energy ratio distribution of the IMF (Δ %), gives the average energy ratio distribution of the IMF that is greater than or equal to the threshold:

(10) $$R_{i}\left[x\right]=\;\{\begin{array}{c} R_{i}\left[x\right],\;\;\;\;R_{i}\left[x\right]\geq \Delta ,\;\;x=m-n\;\;main\;frequency\;band\\ \;\;\;\;0,\;\;\;\;R_{i}\left[x\right]<\Delta ,\;\;\;\;\;\;\;\;\;\;\;\;\;\;\;\;\;\;\;\;\;\;\;\;\;\;\;\;\;\;\;\;\;\;\;\;\;\;\;\;\;\;\;\;\;\;\;\;\;\;\;\;\;\;\;\;\;\;\;\;\;\;\;\;\; \end{array}$$

Then, the EDI is defined as:

(11) $$EDI_{i}=\sum_{x=m}^{n}R_{i}\left[x\right]$$

This shows the cumulative sum of the average energy ratio distribution of the *i*th IMF greater than or equal to the threshold. Then, the EDI can be used to obtain the main frequency band, maximum frequency, and energy density of the signal in each IMF, as follows:

(1) Set the threshold of the average energy ratio distribution of the IMF. The value of the threshold can be set according to the trend of the marginal spectrum, a fixed value or fixed proportion, or by taking the first three maximum frequency bands.
(2) Check if the average energy ratio distribution of the IMF is greater than or equal to the threshold.
(3) Calculate the average energy density intensity, and the main frequency band parameters of the signal energy concentration.

**Figure 4:**
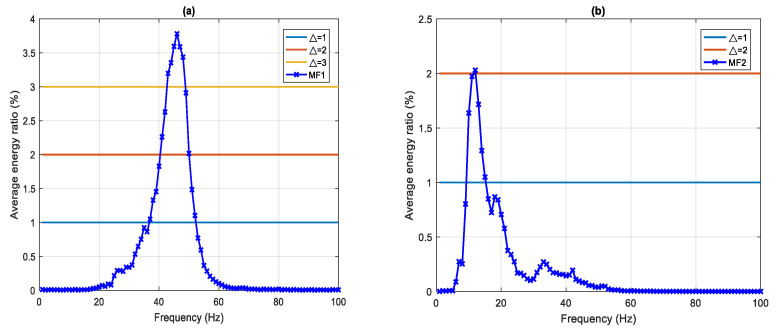
MF of the IMF in 59 samples, average frequency–energy distribution, (**a**) MF1 (**b**) MF2.

Table 2 and Figure 4 show the MF1 and MF2, that is, the MF of the 1st and 2nd IMFs for the 59 samples. For MF1, the average EDI are 39.01%, 30.76%, and 20.95% when the thresholds (Δ) are 1%, 2%, and 3%, and the main frequency bands are 37–52 Hz, 41–50 Hz, and 43–48 Hz, respectively. The maximum frequency and EDI were 46 Hz and 3.78%, respectively, in MF1. For MF2, the average EDI are 9.7% and 2.03%, when the threshold (Δ) is 1% and 2%, and the main frequency bands are 10–15 Hz and 12 Hz, respectively. The maximum frequency and EDI were 12 Hz and 2.03%, respectively. Therefore, following the above process, we have captured the key characteristic parameters of the signal: the energy ratio distribution, EDI, and the main frequency band and the maximum frequency. These will be used to perform the next signal classification step.

(5) Classification

After calculating the energy ratio distribution of each IMF, the criteria are formulated to test for similarity according to the characteristics of this parameter. The classification criteria can be quantified according to the correlation of the energy ratio distribution, such as the same number of IMFs after EMD, the energy ratio of one IMF (usually the first) being much larger than the energy ratio of other IMFs, or multiple IMFs with similar energy ratios.

Furthermore, the sampling time interval can be adjusted to enable more detailed observation, analogous to adjusting the magnification on a microscope. We classified the 59 samples with the same number of IMFs into two categories according to the following classification criteria:

Class I:

(1) The energy ratio of the 1st IMF is much larger than those of the other IMFs, (IMF1 >> IMFs, IMF1 > 70%).
(2) In the MF1 analysis process, the maximum frequency of IMF1 is summarized according to the EDI method. Then the maximum frequencies of all sampling signals are compared to define the classification range of the maximum frequency, (maximum frequency range of 41–45 Hz).

According to the above two classification criteria, there are seven samples in class I.

Class II:

(1) The energy ratio of the 2nd IMF was larger than a fixed number, (IMF2 > 30%).
(2) In the MF2 analysis process, the maximum frequency of IMF2 is summarized according to the EDI method. Then the maximum frequencies of all sampling signals are compared to define the classification range of the maximum frequency, (maximum frequency range 10–14 Hz).

According to the above two classification criteria, there are six samples in class II.

(6) Hilbert Spectrum (HS)

The amplitude size a(*t*) and IF IF*i* (t) of a signal are functions that change over time. For given sampling time intervals and sampling frequency resolution intervals, $\omega_{i}\left(t\right),$ the HS, *H*(ω, t), for each IMF can be generated as follows:

(12) $$\;H_{i}\left(\omega ,\;t\right)=\{\begin{array}{c} a_{i}\left(t\right),\;\;\omega =\omega_{i}\left(t\right)\\ 0,\;\;otherwise \end{array}$$

(13) $$H\left(\omega ,\;t\right)=\overset{N}{\underset{i=1}{\sum }}H_{i}\left(\omega ,\;t\right)$$

After squaring the amplitude size, ${\mathrm{IMF}}_{imnt12}^{2}$(*t,f*), setting the sampling time interval to t_1_–t_2_, and setting the sampling frequency resolution to *m*–*n* Hz, the HS of the *i*th IMF (average time–frequency–energy distribution) becomes:

(14) $$HS_{i}=\frac{{\mathrm{IMF}}_{imnt12}^{2}\left(t,f\right)}{E_{total}}$$

## 3. Analysis Results

(1) Energy analysis

**Figure 5:**
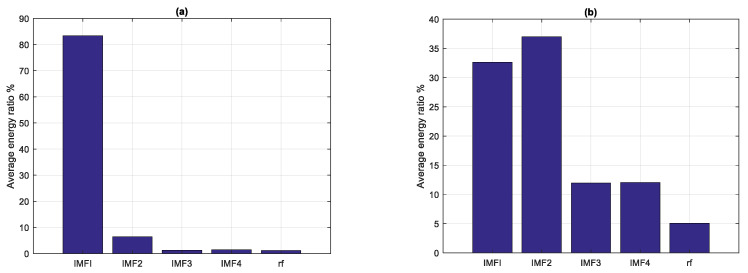
Average energy ratios of IMFs for (**a**) class I and (**b**) class II.

The experiment in this study first classified the 59 samples according to the above criteria. Therefore, the average energy ratios of four IMFs and one RF were calculated. The experimental data are shown in Table 3 and Figure 5. The average energy ratios of the IMFs 1/2/3/4/RF were 83.40%, 6.44%, 1.29%, 1.43%, and 1.21%, respectively, in class I. The average energy ratios of the IMFs 1/2/3/4/RF were 32.63%, 37.00%, 11.95%, 12.07%, and 5.09%, respectively, in class II.

(2) Marginal Frequency (MF)

**Figure 6:**
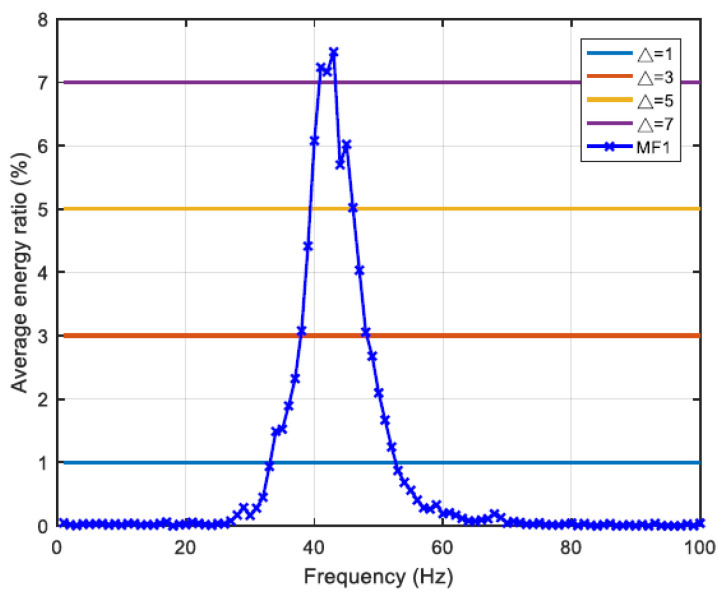
MF1 of class I average frequency–energy distribution.

The MF spectra of class I (seven samples) and class II (six samples) were analyzed with a sampling time interval of 180 ms, and a sampling frequency of 1 Hz. In class I, because the energy ratio of IMF1 is much larger than that of other IMF_*i*_, only MF1 was analyzed. Setting the threshold of the average energy ratio Δ = 1%, 3%, 5%, and 7% gives EDIs of 74.18%, 59.26%, 44.69%, and 21.87%, with the main frequency bands 34–52 Hz, 38–48 Hz, 40–46 Hz, and 41–43 Hz, respectively. The maximum frequency is 43 Hz with a corresponding EDI of 7.48%, as shown in Table 4 and Figure 6.

In class II, because the energy of IMF1 and IMF2 is much greater than that of the other IMF_*i*_, only MF1 and MF2 are analyzed. For MF1, setting the threshold Δ = 1%, 2%, and 3% gives EDIs of 24.08%, 15.17%, and 3.08%, with the main frequency bands at 41–52 Hz, 45–50 Hz, and 49 Hz, respectively. The maximum frequency is 49 Hz with a corresponding EDI of 3.08%. For MF2, setting the threshold Δ = 1%, 2%, 3%, 4%, and 5% leads to EDIs of 28.29%, 22.79%, 18.11%, 15.07%, and 5.59% with the main frequency bands at 10–18 Hz, 10–15 Hz, 10–13 Hz, and 12 Hz, respectively. The maximum frequency is 12 Hz with a corresponding EDI of 5.59% in this case.

**Figure 7:**
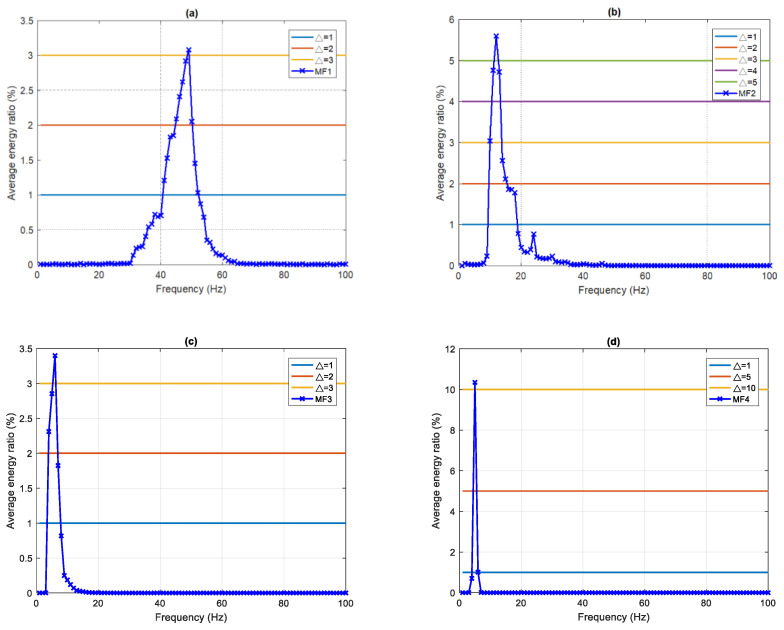
An average frequency–energy distribution of class II (**a**) MF1, (**b**) MF2, (**c**) MF3, and (**d**) MF4.

For MF3, setting the Δ = 1%, 2%, and 3% gives EDIs of 10.38%, 8.56%, and 3.39%, with main frequency bands at 4–7 Hz, 4–6 Hz, and 6 Hz, respectively. The maximum frequency is 6 Hz with a corresponding EDI of 3.39%. For MF4, the chosen threshold values Δ = 1% and 2% lead to EDIs of 11.36% and 10.35%, with main frequency bands at 5–6 Hz, and 5 Hz, respectively. The maximum frequency is 5 Hz with an EDI of 10.35%. This is summarized in Table 5 and Figure 7.

(3) Hilbert Spectrum (HS)

Every 180 ms time interval was divided into six equal length interval signals with a sampling time of 30 ms. Then, the MF spectrum was calculated for each of these signals, and the HS (time–frequency–energy distribution) was obtained. Both class I (seven samples) and class II (six samples) were analyzed with a sampling time interval of 30 ms, and a frequency resolution of 1 Hz. In class I, since the energy ratio of IMF1 is much larger than that of the other IMF_*i*_, only HS1 is analyzed; in class II, since the energy of IMF1 and IMF2 is much greater than that of the other IMF_*i*_, both HS1 and HS2 are calculated.

**Figure 8:**
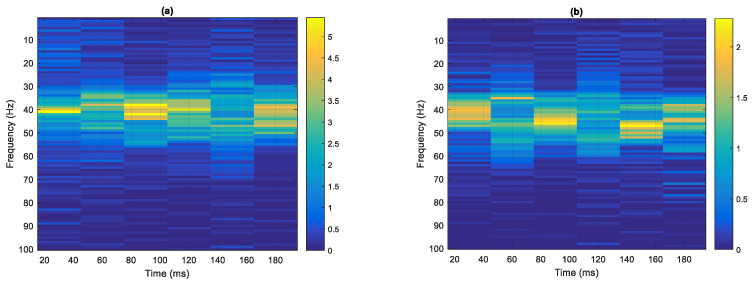

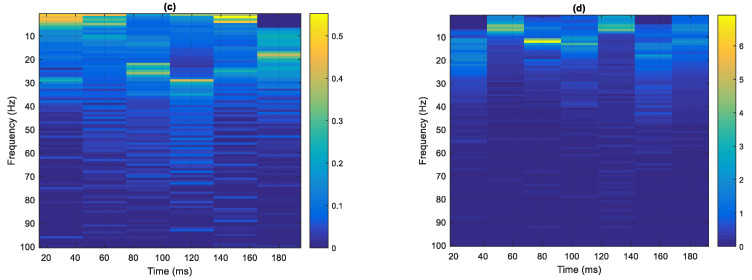
Hilbert Spectrum, time–frequency–energy distribution, (**a**) HS1 of class I, (**b**) HS1 of class II, (**c**) HS2 of class I, and (**d**) HS2 of class II.

The HS1 of class I was calculated using the main frequency band of MF1 with a threshold of 2% (41–50 Hz), and is shown in Table 6 and Figure 8a. The maximum energy ratio (5.3723%, highlighted red in Table 6) occurs at 30 ms and a frequency of 41 Hz. The second-highest energy ratio (5.3772%, highlighted in blue) occurs at 90 ms, and a frequency of 42 Hz.

The results of the calculation of the HS1 of class II are shown in Table 7 and Figure 8b. The maximum energy ratio (2.2794%, highlighted in red in Table 7) occurs at 90 ms, and a frequency of 46 Hz. The second highest energy ratio (2.1943%, highlighted in blue) occurs at 150 ms, and a frequency of 47 Hz.

The HS2 of class I was evaluated using the main frequency band of MF2 with a threshold of 1% (10–15 Hz), which is shown in Table 9 and Figure 8c. The maximum energy ratio (0.2417%, highlighted in red in Table 8) occurs at 180 ms and a frequency of 10 Hz. The second-highest energy ratio (0.2281%, highlighted in blue) occurs at 180 ms and a frequency of 11 Hz.

The results of the calculation of the HS2 of class II, using the same frequency band and threshold as for class I, is shown in Table 9 and Figure 8d. The maximum energy ratio (6.9196%, highlighted in red in Table 9) occurs at a time of 90 ms and a frequency of 12 Hz. The second-highest energy ratio (4.1130%, highlighted in blue) occurs at a time of 90 ms and a frequency of 11 Hz.

## 4. Discussion

The purpose of the energy characteristic classification method proposed in this paper is to use the EMD process to leave signals with high energy and similar characteristics to improve the efficiency of signal feature extraction and data processing. The multi-component signal of the blue whale (B call) is used to perform MF and HS analysis for each IMF after the EMD of each sampling time interval (180 ms). At the same time, the EDI method is used to set the total energy ratio at the appropriate fixed value. From this, the main frequency of the two sets of signals, the maximum frequency, and related characteristic parameters can be obtained. The main frequency bands are distributed at 43–48 Hz in IMF1 when the threshold is 3%, and 10–15 Hz in IMF2 when the threshold is 1%.

**Figure 9:**
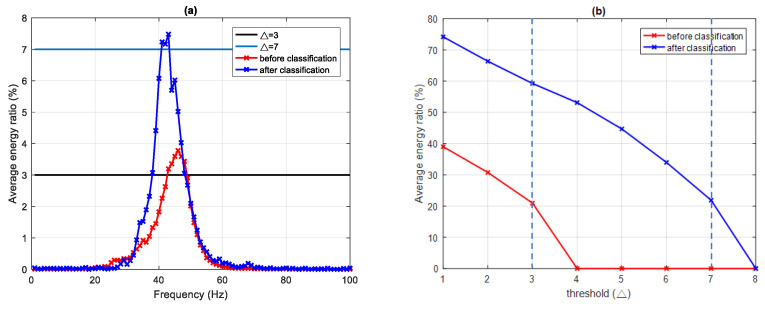
MF1 of class I, (**a**) average frequency–energy distribution comparison of before and after classification with Δ = 3%, and Δ = 7%, and (**b**) threshold and average energy ratio distribution with before and after classification.

After the EDI classification method, the results (see Figure 9) clearly show that the feature extraction from the signal after classification is better than before classification. In the MF1 of class I for the signal before classification, using a threshold of 3% leads to an energy ratio of 20.95% with the main frequency band of 43–48 Hz, and a maximum frequency of 46 Hz. Using the same threshold for the signal after classification gives a much greater energy ratio of 59.26% with a main frequency band of 38–48 Hz. Furthermore, the signal before classification shows no intensity for thresholds ≥4%; conversely, the signal after classification continues to be measurable until the threshold exceeds 7%.

**Figure 10:**
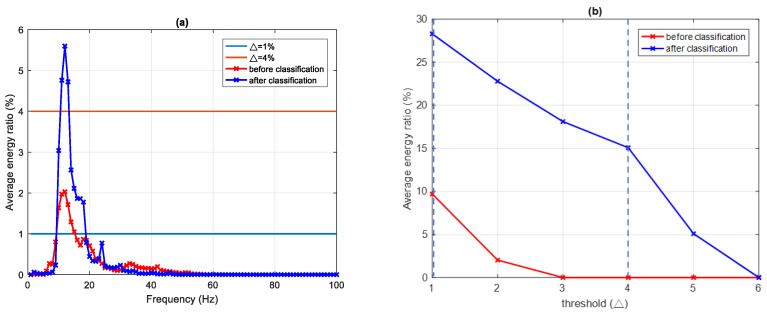
MF2 of class II, (**a**) average frequency–energy distribution comparison of before and after classification with Δ = 1%, and Δ = 4%, and (**b**) threshold and average energy ratio distribution with before and after classification.

Figure 10 shows similar results for the MF2 of class II. For the signal before classification, a threshold of 1% gives an energy ratio of 9.7% with the main frequency band at 10–15 Hz. At the same threshold, the signal after classification gives a greater energy ratio of 28.29% with a main frequency band at 10–18 Hz. Again, a higher threshold can be applied to the signal after classification, which remains measurable up to a threshold of 5% (energy ratio 5.59%) compared to a maximum threshold below 3% for the signal before classification.

Table 10 shows the MF1 and MF2 of the IMFs, average energy intensity density (EDI), main frequency band, max frequency with thresholds for the SWAL’s, Monterey Bay Aquarium Research Institute’s (MBARI), and Ocean Networks Canada’s (ONC) BWBCV. The MBARI’s blue whale B call vocalization (BWBCV) was obtained from the MBARI website [27], and the time length of the second BWBCV is approximately 225 s. Part I is 70 to 90.16 s, and part II is 203 to 222.62 s. A total of 65 and 78 analysis samples with a time duration of 180 ms were extracted from parts I and II of the MBARI’s BWBCV, respectively. The ONC’s BWBCV was obtained from the ONC website [32], the time length of the sampling valid signal is 21.06 s, and 52 analysis samples with a time duration of 180 ms were extracted.

The analysis results show that the high-resolution main frequency band is distributed at 43–48 Hz with a threshold of 3%, and 10–15 Hz with a threshold of 1%, in the MFs of IMF1 and IMF2 for the SWAL’s BWBCV, respectively. The average EDI was 20.95%, and 9.70%, respectively. The maximum frequency of MF1 (the MF of IMF1), and MF2 (the MF of IMF2) was 46 Hz with an EDI of 3.38%, and 12 Hz with an EDI of 2.03%, respectively.

The analysis results also show that the high-resolution main frequency band is distributed at 35–41 Hz with a threshold of 3%, and 31–39 Hz with a threshold of 2%, in the MF of IMF1 for MBARI’s parts I and II BWBCVs, respectively. The average EDI was 26.46%, and 23.59%, respectively. The maximum frequency of MF1 for MBARI’s parts I and II BWBCVs was 38 Hz with an EDI of 4.15%, and 36 Hz with an EDI of 2.96%, respectively.

The analysis results show that the high-resolution main frequency band is distributed at 11–13 Hz with a threshold of 1%, and 9–16 Hz with a threshold of 1%, in the MF of IMF2 for MBARI’s part I and II BWBCVs, respectively. The average EDI was 3.9% and 12.22%, respectively. The maximum frequency of MF1 (the MF of IMF1) for MBARI’s part I and II BWBCVs was 12 Hz with an EDI of 1.4%, and 13 Hz with an EDI of 1.79%, respectively.

The analysis results show that the high-resolution main frequency band is distributed at 27–32 Hz with a threshold of 2%, and 11–14 Hz with a threshold of 2%, in the MFs of IMF1 and IMF2 for the ONC’s BWBCV, respectively. The average EDI was 12.7% and 10.93%, respectively. The maximum frequency of MF1 and MF2 was 30 Hz with an EDI of 2.27%, and 12 Hz with an EDI of 3.15%, respectively.

According to the experimental data analysis in Table 10, the following four phenomena can be observed:

(1) BWBCV is a frequency-modulated tonal that contains fundamental frequency and several harmonics, where MF2 is a fundamental frequency, and its main frequency band and max frequency are stable, and do not vary greatly depending on the difference vocalizations.
(2) MF1 is the high-frequency harmonic signal, shown in a table. The pattern of each source is similar, but the values of its main frequency band and max frequency vary depending on the difference vocalizations.
(3) From the two observations above, it can be understood that each individual, as well as the same type of sound (such as a B call) emitted by the same individual at different times, has its commonality and uniqueness.
(4) Thus, the proposed method can be successfully applied to the different individuals of the same species in the sound signal processing of the characteristic extraction, and as signal detection and classification.

## 5. Conclusions

In this study, a method is proposed for blue whale vocalization sample signal feature extraction analysis. There are six steps in the analysis algorithm process: EMD, energy analysis, MF, FE, classification by EDI, and HS analysis. Three main results are produced.

(1) The energy ratio of the IMF. From this, it is possible to understand the change in signal strength, develop the classification criteria of similarity, and summarize the relevant signal analysis.
(2) The MF and HS spectrograms.
(3) The EDI, which is used to set the threshold of the average energy ratio of the IMF, allows the main frequency band, the maximum frequency, and the percentage of the total energy ratio to be calculated.

Following the process, the blue whale B call sound source signals are divided into two categories (class I and II) for analysis. Through the spectrogram time–frequency–energy distribution and EDI analysis, the first and second IMFs are obtained as a combination of the main signals. The main frequency bands of the first and second IMFs are 43–48 Hz (with Δ = 3%) and 10–15 Hz (with Δ = 1%), respectively, before classification. After classification, the main frequency bands are 41–43 Hz (with Δ = 7%, class I) and 11–13 Hz (with Δ = 4%, class II), respectively. Here, we successfully extracted the signal feature parameters for the BWBCV signals.

The proposed method can be applied in underwater acoustic research involving various marine organisms, such as marine mammals, cetaceans, and dolphins. Subsequently, it could be integrated into signal processing, detection, and classification research fields.

## Figures and Tables

**Table 1:** Average energy ratio of intrinsic mode function IMF1/2/3/4/RF (59 samples with 4*IMF + 1*RF).

IMF_*i*_	IMF1	IMF2	IMF3	IMF4	*rf*
Average energy ratio (%)	49.06	20.58	13.51	10.94	3.84

**Table 2:** MF1 and MF2 of the IMF in 59 samples, EDI, main frequency band, max frequency with thresholds of 1, 2, and 3%.

MF1 of 59 Samples			MF2 of 59 Samples		
Δ	EDI(%)	Main Frequency (Hz)	Δ	EDI(%)	Main Frequency (Hz)
1	39.01	37~52	1	9.7	10~15
2	30.76	41~50	2	2.03	12
3	20.95	43~48	20.3	2.03	12(max freq.)
3.78	3.78	46(max freq.)			

**Table 3:** Average energy ratios of class I/II with IMF1/2/3/4/*RF*.

%	ENGIMF1	ENGIMF2	ENGIMF3	ENGIMF4	ENG*rf*
Class I	83.4	6.44	1.29	1.43	1.214
Class II	32.63	37	11.95	12.07	5.09

**Table 4:** The MF1 of class I, EDI, main frequency band, and maximum frequency for threshold Δ = 1%, 3%, 5%, and 7%.

MF1 of Class I		
Δ	EDI (%)	Main Frequency (Hz)
1	74.18	34~52
3	59.26	38~48
5	44.69	40~46
7	21.87	41~43
7.48	7.48	43(max freq.)

**Table 5:** MF1, MF2, MF3, and MF4 of class II, EDIs, main frequency bands, and the maximum frequency for threshold values of 1%, 2%, 3%, 4%, and 5%.

MF1 of Class II			MF2 of Class II			MF3 of Class II			MF4 of Class II		
Δ	EDI (%)	Main Frequency (Hz)	Δ	EDI (%)	Main Frequency (Hz)	Δ	EDI (%)	Main Frequency (Hz)	Δ	EDI (%)	Main Frequency (Hz)
1	24.08	41~52	1	28.29	10~18	1	10.38	4~7	1	11.36	5~6
2	15.17	45~50	2	22.79	10~15	2	8.56	4~6	2	10.35	5
3	3.08	49	3	18.11	10~13	3	3.39	6	10.35	10.35	5(max. feq.)
3.08	3.08	49(max. feq.)	4	15.07	11~13	3.39	3.39	6(max. feq.)			
			5	5.59	12						
			5.59	5.59	12(max. feq.)						

**Table 6:** The data of HS1 of class I, main frequency band 41–50 Hz, sampling time interval 30 ms. The maximum and second-highest energy ratios are highlighted in red and blue, respectively.

Freq. (Hz)/ Time (ms)	41	42	43	44	45	46	47	48	49	50
30	5.3772	2.7042	2.0189	1.6313	2.2850	1.3131	1.4670	1.8087	1.1427	0.9644
60	1.8089	2.3290	2.5002	2.2260	1.5188	2.5957	2.2484	2.9589	1.5979	0.6230
90	4.2118	5.3723	3.9669	4.3410	1.6975	1.3459	1.7999	1.7419	2.1899	1.6947
120	3.2341	2.5195	3.1334	2.8402	2.9004	2.8112	2.8166	1.5609	2.4858	1.8153
150	1.3895	1.4866	1.5857	3.0558	2.3109	1.6275	3.2360	2.4181	2.5619	1.9033
180	4.0709	3.6968	3.3743	2.7741	3.5424	3.6585	4.0669	2.6698	2.2187	3.2907

**Table 7:** The data of HS1 of class II, main frequency band 41–50 Hz, sampling time interval 30 ms. The maximum and second-highest energy ratios are highlighted in blue and red, respectively.

Freq. (Hz)/ Time (ms)	41	42	43	44	45	46	47	48	49	50
30	1.8098	1.6628	1.6385	1.8475	1.3740	1.1829	1.4319	0.8178	0.5399	0.1937
60	0.6000	0.7085	0.5411	1.1298	1.3377	1.2971	1.0400	1.0035	1.0035	0.8401
90	1.2738	1.4977	1.5970	1.9975	2.1943	1.4831	1.3497	1.1374	1.1374	1.1350
120	1.2376	0.7775	0.7527	1.0351	0.7279	0.7795	0.4897	0.8161	0.8161	0.8087
150	0.5638	0.6451	0.6744	0.7585	2.0259	2.2794	1.7586	1.2902	1.2902	1.7578
180	1.2230	0.8972	1.0387	1.7696	0.9117	1.3053	1.2976	1.0124	1.0124	0.7057

**Table 8:** The data of HS2 of class I, main frequency band 10–15 Hz, sampling time interval 30 ms. The maximum and second-highest energy ratios are highlighted in red and blue, respectively.

Freq. (Hz)/ Time (ms)	10	11	12	13	14	15
30	0.1397	0.1436	0.1450	0.1035	0.0915	0.1032
60	0.2132	0.1826	0.1443	0.1220	0.1377	0.0893
90	0.1161	0.1367	0.1169	0.1595	0.1507	0.1042
120	0.0924	0.0873	0.0853	0.0839	0.0665	0.0642
150	0.0994	0.0929	0.0608	0.0772	0.0666	0.1291
180	0.2417	0.2281	0.1939	0.2113	0.2132	0.1520

**Table 9:** The data of HS2 of class II, main frequency band 10–15 Hz, sampling time interval 30 ms. The maximum and second-highest energy ratios are highlighted in red and blue, respectively.

Freq. (Hz)/ Time (ms)	10	11	12	13	14	15
30	0.6355	1.9432	2.0005	1.3782	1.7531	1.7134
60	0.8390	0.7247	0.6159	0.5713	0.3830	0.3698
90	0.8440	4.1130	6.9196	2.2401	1.3338	0.9931
120	1.9624	2.2184	1.5042	3.6569	1.5626	1.8535
150	0.3579	0.3781	0.3199	0.3984	0.3454	0.3895
180	0.8606	0.6916	0.6527	1.6893	1.6208	1.6371

**Table 10:** MF1 and MF2 of the IMFs, average EDI, main frequency band, max frequency with thresholds for the SWAL’s, MBARI’s and ONC’s BWBCVs.

Source (Blue Whale B Call)	Sampling Valid Signal (sec)	Number of Sampling (4*IMFs & 1*RF)	Average Energy Distribution of IMF1/IMF2	Main Frequency of MF1 (Δ, EDI%)	Max. Frequency of MF1	Main Frequency of MF2 (Δ, EDI%)	Max. Frequency of MF2
Scripps Whale Acoustic Lab	16.92	59	49.06/20.58	43–48 Hz (Δ = 3, 20.95%)	46 Hz (EDI% = 3.38%)	10~15 Hz (Δ = 1, 9.7%)	12 Hz (EDI% = 2.03%)
Monterey Bay Aquarium Research Institute (part I)	20.16	65	57.13/14.97	35–41 Hz (Δ = 3, 26.46%)	38 Hz (EDI% = 4.15%)	11~13 Hz (Δ = 1, 3.9%)	12 Hz (EDI% = 1.79%)
Monterey Bay Aquarium Research Institute (part II)	19.62	78	50.81/20.69	31–39 Hz (Δ = 2, 23.59%)	36 Hz (EDI% = 2.96%)	9~16 Hz (Δ = 1, 12.22%)	13 Hz (EDI% = 1.79%)
Ocean Networks Canada	21.06	52	52.2/24.5	27–32 Hz (Δ = 2, 12.7%)	30 Hz (EDI% = 2.27%)	11~14 Hz (Δ = 2, 10.95%)	12 Hz (EDI% = 3.15%)

## Data Availability

Not applicable.

## Abbreviations

BWBCV	blue whale B call vocalization
EDI	energy density intensity
EMD	empirical mode decomposition
FE	feature extraction
FFT	fast Fourier transform
HS	Hilbert spectrum
HT	Hilbert transform
HHT	Hilbert Huang transform
IF	instantaneous frequency
IMF	intrinsic mode function
MBARI	Monterey Bay Aquarium Research Institute
MF	marginal frequency
ONC	Ocean Networks Canada
RF	residue function
SNR	signal to noise ratio
SWAL	Scripps Whale Acoustics Lab
TFD	time–frequency distribution functions
WT	wavelet transform
